# Hereditary cancer risk assessment: essential tools for a better approach

**DOI:** 10.1186/1897-4287-11-16

**Published:** 2013-10-28

**Authors:** Israel Gomy, Maria Del Pilar Estevez Diz

**Affiliations:** 1 Instituto do Câncer do Estado de São Paulo da Faculdade de Medicina da Universidade de São Paulo, Av. Dr. Arnaldo, 251, Cerqueira César, São Paulo, Brazil

**Keywords:** Germline, Susceptibility, Genetic testing, Hereditary cancer, Counseling

## Abstract

Hereditary cancer risk assessment (HCRA) is a multidisciplinary process of estimating probabilities of germline mutations in cancer susceptibility genes and assessing empiric risks of cancer, based on personal and family history. It includes genetic counseling, testing and management of at-risk individuals so that they can make well-informed choices about cancer surveillance, surgical treatment and chemopreventive measures, including biomolecular cancer therapies. Providing patients and family members with an appropriate HCRA will contribute to a better process of making decisions about their personal and family risks of cancer. Following individuals at high risk through screening protocols, reassuring those at low risk, and referring those at increased risk of hereditary cancer to a cancer genetics center may be the best suitable approach of HCRA.

## Introduction

Within the last decade, emerging biomolecular technologies, such as whole-exome and genome sequencing and high-throughput genotyping have been rapidly growing and enlightening the knowledge of inherited cancer susceptibility. Meanwhile, important issues on bench-to-bedside translation of these major breakthroughs into clinical practice have been equally addressed. Nevertheless, one essential component of these aspects includes the counseling of individuals with hereditary cancer risk. The increased public awareness of the genetic aspects of cancer susceptibility has resulted in more enquiries from clinical and surgical oncologists about which would be the best approach for their patients so that appropriate management could be provided.

More than fifty rare Mendelian cancer syndromes are caused by highly penetrant germline mutations affecting either tumor suppressor genes, DNA repair genes or proto-oncogenes, mostly with autosomal dominant inheritance (Table 1). Their cumulative relative risks are the highest ones (more than 10 times), dramatically affecting the quality of life and decreasing its expectancy. Moderately penetrant germline mutations are also rare, although more prevalent in selected populations, and increase approximately two to five times the general population risk. Germline variants with low penetrance are relatively common in most populations, and have been identified through genome-wide association studies (GWAS). Although their size effects are individually low (< 1.5), they may be relevant on risk stratification. Actually, they explain part of the excess familial risk and the so-called 'missing heritability’ remains largely unknown [1]. Nowadays, with the advance of next-generation sequencing and genotyping assays, more variants have been identified, shedding new light on the genomic architecture of the inherited susceptibility of cancer.

**Table 1:** Hereditary cancer syndromes

**Syndrome**	**Gene**	**Mutation status**	**Penetrance**	**Tumors**
Hereditary breast and/or ovarian cancer	*BRCA1*	Heterozygous	High	Breast cancer
	*BRCA2*			Ovarian cancer
	*RAD51 (B,C,D)*		intermediate	Pancreatic cancer
	*ATM*		intermediate	Prostate cancer
	*CHEK2*		intermediate	Colorectal cancer
Lynch syndrome	*MLH1*	Heterozygous	High	Colorectal cancer
	*MSH2*			Endometrial cancer
	*MSH6*			Ovarian cancer
	*PMS2*			Gastric cancer
	*EPCAM*			
MMR cancer syndrome	MMR genes	Homozygous	High	Leukemia, lymphoma
				Rhabdomyosarcoma
Familial adenomatous polyposis	*APC*	Heterozygous	High	Gastrointestinal adenomas
				Colorectal cancer
				Duodenal cancer
MYH-associated polyposis	*MUTYH*	Homozygous	High	Colorectal cancer
Polymerase proofreading-associated polyposis	*POLE*	Heterozygous	High	Colorectal cancer
	*POLD1*			Endometrial cancer
Bloom syndrome	*BLM1*	Homozygous	High	Leukemia
				Colorectal cancer
				Wilms tumor
Nijmegen syndrome	*NBS1*	Homozygous	High	Lymphoma
				Medulloblastoma
				Rhabdomyosarcoma
Fanconi anemia	*FANC* genes (includes *BRCA2, PALB2, BRIP1*)	Homozygous	High	Leukemia
				Medulloblastoma
				Wilms tumor
Li-Fraumeni syndrome	*TP53*	Heterozygous	High	Breast cancer
Li-Fraumeni like syndrome	*CHEK2*		intermediate	Sarcoma
				Adrenocortical cancer
				Brain tumor
Cowden syndrome	*PTEN*	Heterozygous	High	Hamartomatous polyps
				Skin tumors
				Breast cancer
				Thyroid cancer
				Endometrial cancer
Hereditary diffuse gastric cancer	*CDH1*	Heterozygous	High	Diffuse gastric cancer
				Lobular Breast cancer
Peutz-Jeghers syndrome	*STK11*	Heterozygous	High	Hamartomatous polyps
				Colorectal
				Small bowel
				Breast cancer
				Pancreatic cancer
Juvenile polyposis	*SMAD4*	Heterozygous	High	Hamartomatous polyps
	*BMPR1A*			Colorectal cancer
				Pancreatic cancer
Melanoma syndromes	*CDKN2A*	Heterozygous	High	Malignant melanoma
	*CDK4*			Pancreatic cancer
Neurofibromatosis	*NF1*	Heterozygous	High	Vestibular schwannoma
	*NF2*			Meningioma
				Neurofibroma
				Optic glioma
Tuberous sclerosis	*TSC1*	Heterozygous	High	Renal angiomyolipoma
	*TSC2*			Subependimoma
				Giant cell astrocytoma
Von Hippel-Lindau syndrome	*VHL*	Heterozygous	High	Hemangioblastomas
				Renal cell cancer
				Pheochromocytoma
Chuvash policitemia		Homozygous	High	Vertebral angiomas
Birt-Hogg-Dubè syndrome	*FLCN*	Heterozygous	High	Renal cell cancer
				Skin tumors
Papillary renal cancer syndromes	*FH*	Heterozygous	High	Renal cell cancer
	*MET*			
Retinoblastoma	*RB1*	Heterozygous	High	Retinoblastoma
Hereditary Paraganglioma	*SDH (A, B, C, D)*	Heterozygous	High	Paraganglioma
				Pheochromocytoma
Multiple Endocrine Neoplasia1	*MEN1*	Heterozygous	High	Pituitary adenoma
Multiple Endocrine Neoplasia2	*RET*			Parathyroid adenoma
				Medular thyroid cancer
				Pheochromocytoma

This review aims to describe a high-quality approach of delivering hereditary cancer risk assessment (HCRA) within a multidisciplinary context.

## Referrals for HCRA

In addition to age, a positive family history of cancer is the single most important constitutional risk factor for which early recognition and intervention could be lifesaving.

Identifying the inherited risk factors of cancer in a given individual or family is complex and raises important psychological, social, and ethical issues. It requires the knowledge of genetics and oncology, besides suitable communication skills, demanding more time than from most clinical services. The American Society of Clinical Oncology (ASCO), the National Society of Genetic Counselors (NSGC), the Oncology Nursing Society (ONS), and other health care professional organizations have set forth guidelines outlining standards for the practice of cancer risk counseling, risk assessment, and genetic testing [2-4]. It also includes genetic testing as appropriate and management of at-risk individuals so that they can make informed choices about cancer screening, surgical and/or chemopreventive risk management options, as well as targeted cancer therapies [5]. In Table 2 there are possible indications of referral for HCRA.

**Table 2:** Possible indications of referrals for hereditary cancer risk assessment

**Personal history**	**Family history**
**Early onset of cancer diagnosis (e.g. breast cancer < 45 years, colorectal cancer < 50 years)**	Three close relatives (same side) with cancer of the same or syndromically related type (breast/ovary, colorectal/endometrium)
**Multiple associated primary cancers: breast/ovary, colorectal/endometrium**	Two close relatives (same side) with cancer of the same or related type with at least one affected under 50 years
**Male breast cancer**	One first-degree relative with early onset cancer (breast < 45 years, colorectal < 50 years)
**Ovarian, fallopian tube, primary peritoneal cancer**	One first-degree relative with multiple primary cancers
**Breast cancer and thyroid, sarcoma, adrenocortical carcinoma**	Two or more relatives with uncommon cancers (sarcoma, glioma, hemangioblastoma, etc.)
**Multiple colon polyps (>10 cumulative)**	Relatives of patients with known *BRCA, APC, MUTYH*, mismatch repair mutations
**Colorectal or endometrial cancer with microsattelite instability and/or lack of expression of mismatch repair protein(s) by immunohistochemistry**	Many relatives with cancer but no criteria for testing are fulfilled

## Components of the HCRA

The genetic risk assessment of an individual with cancer is based upon the careful analysis of the personal history, detailed family history and physical examination when appropriate. It requires confirmation of the diagnosis in affected relatives, preferably through biopsies or, whenever possible, death certificates or autopsies.

These are some important issues to be addressed within the HCRA process [5]:

1. Family pedigrees drawings with at least three generations in both sides of family;

2. Patients and relatives:

a. Current age, age at diagnosis, age at death, primary site, pathologic features, treatments;

a. Ancestry (especially if Ashkenazi Jewish);

a. Previous surgeries, biopsies, diseases;

a. Endogenous risk factors: age at menarche, fertility history;

a. Exogenous risk factors: tobacco/alcohol use, food intake, hormones, exercises;

a. Cancer screening: mammography, gastrointestinal endoscopy, PSA;

a. Chemoprevention.

3. Physical examination (when appropriate): skin, head circumference, tongue, oral mucosa, thyroid, hands and feet, abdomen;

4. Psychosocial and family dynamic;

5. Basic principles of cancer genetics;

6. Differential diagnosis;

7. Mutation probabilities and empiric risks;

8. Pre-test genetic counseling:

a. Indentify the best individuals to test;

a. Prioritize order of testing (germline, tumor);

a. Explain test techniques, limitations, sensitivity/specificity;

a. Facilitate informed consent: possible outcomes (positive, true-negative, uninformative), costs, turn-around-time, insurance coverage;

a. Address psychological, ethical, cultural, communication issues.

9. Post-test genetic counseling:

a. Disclosure and interpretation of results;

a. Address psychological and ethical concerns;

a. Identify at-risk family members;

a. Discuss communication of results to at-risk family members.

10. Personalized risk management strategies:

a. Screening/surveillance exams;

a. Risk reduction, cancer prevention (surgeries, chemoprevention);

a. Empiric strategies for uninformative results.

## Predicting germline mutations

Several models are available to estimate the likelihood of detecting a mutation in a cancer-susceptibility gene and each model is utilized selectively based on the characteristics of the patient’s personal and family history.

If a mutation in the BRCA gene is suspected to be present in a hereditary breast and ovarian cancer family, there are several models available to predict the probability of an individual carrying such a mutation. These models include the Couch [6], Penn II [7], Myriad [8], BRCAPRO [9-11], Tyrer-Cuzick [12], and BOADICEA models [13]. Such models incorporate breast and ovarian cancer in first- and second-degree relatives, age of onset of cancer, Ashkenazi Jewish ancestry, and some are starting to incorporate other ethnic backgrounds.

There are similar models for predicting mutation in DNA mismatch repair (MMR) genes in suspected Lynch syndrome families, including Wijnen [14], MMRpro [15], MMRpredict [16], and PREMM1,2,6 [17]. However, in the HCRA of colon cancer families, it is more common to use established criteria as an indication for testing, including the Amsterdam I and II [18], or the revised Bethesda Guidelines [19], which determine eligibility for tumor analysis to detect microsatellite instability that would aid to guide genetic testing of the MMR genes.

Furthermore, there are established diagnostic criteria for Li-Fraumeni [20,21] and Cowden syndromes [22,23], as well as mutation probability models for hereditary melanoma [24] and pancreatic cancer (Table 3).

**Table 3:** Clinical criteria guidelines and mutation probability models utilized for HCRA

**Syndrome**	**Model**	Genes included*	Clinical criteria/tumors included*
Hereditary breast and ovarian cancer	Couch, Penn II, Myriad, Tyrer-Cuzick, BRCAPRO, BOADICEA	BRCA1	Breast cancer (age<45, two primaries, male)
		BRCA2	
			Ovarian cancer
			Pancreatic cancer
			Prostate cancer
			Ashkenazi ancestry
			Family history (one side)
Lynch	Wijnen, MMRpro, MMRPredict, PREMM1,2,6	MLH1	Amsterdam I and II
		MSH2	Bethesda (revised)
		MSH6	Colorectal cancer
			Endometrial cancer
Li-Fraumeni and Li-Fraumeni like	none	none	Classic
			Eeles
			Chompret (revised)
Cowden	Cleveland clinic	PTEN	International Cowden Consortium
			Thyroid, oral papillomas, acral keratoses, skin lipomas, trichilemmomas, uterine leiomyomas, gastrointestinal polyps, dysplastic cerebellar gangliocytoma, fibrocystic breast disease
Hereditary melanoma	MELApro	CDKN2A	Melanoma

*included in mutation probability models.

The use of mutation predictability models is important for several reasons. First, calculating the likelihood of a germline mutation can help clinicians determine which family member is the best candidate for testing. Second, due to the high cost of genetic testing, numerical calculations of mutation probability may provide supportive evidence for insurance companies. Third, for psychosocial reasons, patients who are informed with a numerical estimation of a mutation may have more realistic expectations about the possibility of a positive result. Finally, for worried patients with a low probability of carrying a mutation, and for those with mutations without any influence in gene expression or stability, numerical presentations may provide substantial reassurance regarding screening guidelines based on empiric cancer risks. A recent study highlighted possible health benefits and the cost-effectiveness of primary genetic screening for Lynch syndrome in the general population [25].

Nevertheless, several models may underestimate mutation probability in certain situations such as a limited family structure or specific tumor characteristics [26]. Thus, probabilities predicted by a model must be interpreted in the context of an individual’s personal and family history. The National Comprehensive Cancer Network (NCCN) in USA publishes guidelines annually in order to help clinicians to select which patients are appropriate candidates for either genetic referral or genetic testing [27,28].

## Predicting cancer risks

In the absence of an identified gene mutation, counseling unaffected individuals about their empiric risk of cancer requires careful consideration of the patient’s personal and family history.

Most risk estimates for cancer development are empirical, based on the probability of a genetic component in the individual, and this risk estimate increases if the proband has several affected relatives on the same side of family with the same or related cancers, multiple or early onset cancers, or if the individual has clinical features of a hereditary cancer syndrome [29].

For breast cancer, there are several models that estimate empiric risks, including the Gail [30], Claus [31], BRCAPRO [9-11], Tyrer-Cuzick [12] and BOADICEA [13] models. All of these models incorporate first-degree relatives with breast cancer along with hormone risk factors, although they may vary in which known breast cancer risk factors are incorporated. There are also some published tools available to assess risks for colon, ovarian, lung and melanoma, but few are validated [32]. However, for prostate cancer, whose familial risk is known to be associated with dozens of low-penetrant variants [33], there is not a genetic model so far. Predictability based on the number of affected relatives, degree of consanguinity and also ancestry could be calculated by mathematical models to estimate empiric risks and possibly guide recommendations for appropriate screening. The undergoing discovery of functional alleles reinforces the need for new models to incorporate them better address cancer risk assessment.

Moreover, because those models that convert genotypes into absolute risks are empirically derived, prospective research is needed to confirm the accuracy of these predictions and evaluate the effectiveness of interventions based on individual genetic testing. For instance, the National Institute for Health and Care Excellence (NICE) in the United Kingdom recently released guidelines concerning risk assessment and management of familial breast cancer, even when testing is not performed [34].

How should risks be communicated? They can be given as cancer risk per year, or before a certain age, or within a decade, or as an overall lifetime risk in comparison with the population risk in terms of relative risks. The individual perception of cancer risk should be assessed, as should its possible effects on the health and lifestyle behaviors.

## Pre-test counseling

After establishing risks of identifying a pathogenic germline mutation in a family and indicating the best candidates to be tested, it follows the information process of pre-test genetic counseling, which requires informed consent for testing for a mutation in a cancer susceptibility gene (see list below). It explains the eventual limitations of testing, its possible results, the emotional impact that may arise, and its relevance for employment and insurance. This approach must be nondirective, letting patients make their own decisions after knowing all possible scenarios [35]. In some circumstances, a pathogenic mutation will not be identified but genetic variants with unknown clinical significance, requiring further testing and reclassification. When a known deleterious mutation is detected in a family member, and when the affected individual agrees to release his/her results to the family, predictive testing can be offered to at-risk relatives. Predictive testing often requires two pre-test counseling interviews with up to three months between them, when family, emotional, employment and insurance issues are discussed, as well inheritance and penetrance of the mutation are explained. Screening and preventive options should also be discussed and it must be stressed that no surveillance guideline is flawless, so individuals must bear in mind that abnormal symptoms should never be ignored between screening exams [36].

### Basic elements of informed consent for testing cancer susceptibility genes

1. information on the specific mutation(s) being tested, including whether the range of risk associated with the variant will impact medical care;

2. implications of a positive and negative result;

3. possibility that the test will not be informative;

4. options for risk estimation without genetic testing;

5. risk of passing a genetic variant to children;

6. technical accuracy of the test including where required by law, licensure of the laboratory;

7. fees involved in testing and counselling;

8. psychological implications of test results (benefits and risks);

9. risks and protections against genetic discrimination by employers or insurers;

10. confidentiality issues, including policies related to privacy and data security;

11. possible use of DNA testing samples in future research;

12. options and limitations of medical surveillance and strategies for prevention after genetic testing;

13. importance of sharing genetic test results with at-risk relatives so that they may benefit from this information;

14. plans for follow-up after testing.

## Post-test counseling

When an affected or unaffected patient chooses to undergo testing, post-test counseling helps individuals to interpret and understand their results, whether positive, negative, undetermined or inconclusive. Psychological support may be provided as ambiguity and uncertainty may arise [35]. For example, individuals with a low-risk result may suffer from “the survivor guilt”. High-risk individuals may need to explain positive results to their at-risk closest relatives and counselors (or geneticists) can help sharing this information. It should be emphasized that individuals who have had cancer may be psychologically affected and feel guilty by knowing their pathogenic germline mutation may be passed on through their offspring. In addition, since early diagnosis of cancer improves outcome, a clear protocol for surveillance and possible prophylactic measures must be offered to those at greater risks, even if they refuse or could not afford genetic testing. For example, risk-reducing bilateral mastectomy when treating unilateral breast cancer in a *BRCA1/BRCA2* mutation carrier or hysterectomy and bilateral salpingo-ooforectomy in a *MSH6* mutation carrier. However, the application of such a system requires robust audit of outcomes, both related to cancer morbidity and mortality, and of psychological effects. Establishing a threshold level of risk at which to offer screening is needed in order to assess outcome. Those at moderately increased risk must engage in surveillance strategies whose specificity, sensitivity and cost-effectiveness should be addressed in the long term [36].

## Clinical sequencing

Very recently, the American College of Medical Genetics and Genomics (ACMG) published a policy statement on recommendations for reporting incidental findings–those not related to the primary indication for testing but with potential medical utility–in clinical exome and genome sequencing. This working group presented a “minimum list” of Mendelian disorders for which any known pathogenic and expected pathogenic secondary variants would be routinely reported to the clinician who ordered clinical sequencing. The ACMG estimated that about 1% of sequencing results would include an incidental finding from this list. Remarkably, 16 of 24 disorders (67%) are hereditary cancer syndromes and 13/16 (81%) may be manifested during childhood. As recommended, these variants would be reported independently from patients’ preferences and age, and the ordering physician would be responsible for providing patients and family members with pre-, post test counseling and follow up. The informed consent guidelines for this clinical context are expected to be released soon [37].

## Conclusions

In summary, HCRA is an information process of estimating probabilities of germline mutations in cancer susceptibility genes and assessing empiric risks of cancer based on personal and family history in order to offer molecular diagnosis and clinical management. Providing patients with pre-and post-test genetic counseling can help them to achieve a better informed decision making. Following individuals at high risk with surveillance protocols (such as from NCCN and NICE), reassuring those at low risk, and referring those at increased risk of a hereditary cancer (whether carriers of primary or secondary variants) to a cancer genetics center with multidisciplinary outpatient clinics may allow the best suitable approach of HCRA. Specialized nurses can be settled in district hospitals to undertake pedigrees and risk assessment so they can refer individuals at moderately increased risk for surveillance, those at highly increased risk to the local cancer genetics center and reassure those at low risk.

In Brazil, a National Familial Cancer Network has been built in order to provide families with hereditary cancer a prompt access to diagnosis, management and counseling of the most common hereditary cancer syndromes in a public health care setting [38].

Collaboration with associations of patients and non-governmental foundations would be extremely helpful to provide families with a better support and care.

## Competing interests

The authors declare that they have no competing interests.

## Authors’ contributions

IG designed and wrote the manuscript. MPED supervised the manuscript. Both authors read and approved the final manuscript.
